# Supplementary material for: The genome of the endangered Macadamia jansenii displays little diversity but represents an important genetic resource for plant breeding
Source: Plant Direct. 2021 Dec 14;5(12):e364. doi: 10.1002/pld3.364 (PMC8671617; doi:10.1002/pld3.364)
Supplement: Supplementary file 1 — Table S1: Size of each scaffold and number of genes per scaffold Table S2: Topologically Associated Domains (TADs) analysis summary Table S3: TAD statistics at different resolutions Table S4: Location of cyanogenic genes on pseudo‐molecules Table S5: Location of fatty acid genes on pseudo‐molecules Table S6: SNP heterozygosity statistics in eight Macadamia jansenii accessions Table S7: Polymorphic sites in up to seven of the eight Macadamia jansenii accessions Table S8: Genotype‐specific unique polymorphic SNP sites Table S9: List of duplicated genes in M. jansenii genome, using MCScanX tool Figure S1: Linkage density histogram of Hi‐C assembly of M. jansenii genome Figure S2: BLAST2GO sequence similarity search Figure S3: Gene ontology (GO) analysis by BLAST2GO Figure S4: Frequency graph of AED scores Figure S5: Alignment of the vicilin‐like antimicrobial‐peptide transcript from M. integrifolia and M. jansenii Figure S6: Alignment of anti‐microbial CDS sequence of M. integrifolia against the M. jansenii transcript sequence Figure S7: Ks plot of M. jansenii was generated by Wgd tool. [file PLD3-5-e364-s003.docx]

**Supplementary tables:**

Table S1: Size of each scaffold and number of genes per scaffold.

| Pseudo-molecule | Size of scaffold | No. of genes per scaffold (Total: 31,591) |
| --- | --- | --- |
| Scaffold # 1 | 67682215 | 2302 |
| Scaffold # 2 | 63669590 | 2089 |
| Scaffold # 3 | 58143993 | 2309 |
| Scaffold # 4 | 56076407 | 2156 |
| Scaffold # 5 | 55220784 | 2154 |
| Scaffold # 6 | 53595462 | 2154 |
| Scaffold # 7 | 52077970 | 2190 |
| Scaffold # 8 | 49563658 | 2291 |
| Scaffold # 9 | 49085581 | 2081 |
| Scaffold # 10 | 48974653 | 2229 |
| Scaffold # 11 | 47698009 | 2120 |
| Scaffold # 12 | 46713600 | 2117 |
| Scaffold # 13 | 45610911 | 2144 |
| Scaffold # 14 | 45288529 | 2227 |
| Total | 739.40 MB | 30563 |
| Total length of assembly | 758.43 Mb |  |
| Total genes found | 31,591 |  |
| % of genome length in 14 pseudomolecules | 97.5% |  |
| % of genes in 14 pseudo-molecules | 96.7% (30563) |  |

Table S2: Topologically Associated Domains (TADs) analysis summary

| Feature | Count |
| --- | --- |
| A Compartments | 37 (49.77%) |
| B Compartments | 38 (49.36%) |
| TADs at 10 kbp resolution | 13 |
| TADs at 25 kbp resolution | 84 |
| TADs at 50 kbp resolution | 76 |
| Isochores | 6,473 |
| CTCF Binding Sites | 2,423 |

Table S3: TAD statistics at different resolutions

| Resolution (kbp) | Number of TADs | Mean TAD Size (bp) | Basepairs in TADs (kbp) | Percent genome contained in TADs |
| --- | --- | --- | --- | --- |
| 10 | 13 | 348,461 | 45 | 0.61% |
| 25 | 84 | 497,619 | 418 | 5.65% |
| 50 | 76 | 1,553,947 | 1,123 | 15.19% |

Table S4: Location of cyanogenic genes on pseudo-molecules (Supplied as separate excel sheet).

Table S5: Location of fatty acid genes on pseudo-molecules (Supplied as separate excel sheet).

**Table S6:** SNP heterozygosity statistics in eight *Macadamia jansenii* accessions

| **Accession ID** | **Polymorphic variants** | | | | | | | | **Variant SNP sites** | | | **Heterozygosity^3^** |
| --- | --- | --- | --- | --- | --- | --- | --- | --- | --- | --- | --- | --- |
|  | **Total** | **Replacements** | **MNV** | **Insertions** | **Deletions** | **Total SNP** | **SNP Homozygous^1^** | **SNP Heterozygous^2^** | **Homozygous SNPs** | **Heterozygous SNPs** | **Total** |  |
| 1005* | 5,393,188 | 7,642 | 158,763 | 287,946 | 198,900 | 4,739,937 | 4019** | 4,735,918 | 4,019** | 2,428,956 | 2,432,975 | 0.31 |
| 1161004 | 5,311,865 | 6,534 | 129,887 | 218,716 | 158,864 | 4,797,864 | 784,323 | 4,013,541 | 784,323 | 2,038,553 | 2,822,876 | 0.26 |
| 1161003 | 6,649,485 | 10,754 | 184,115 | 320,586 | 235,055 | 5,898,975 | 1,047,938 | 4,851,037 | 1,047,938 | 2,465,089 | 3,513,027 | 0.32 |
| 1161005 | 6,109,728 | 9,908 | 175,182 | 307,401 | 224,149 | 5,393,088 | 780,593 | 4,612,495 | 780,593 | 2,347,362 | 3,127,955 | 0.3 |
| 1161001a | 6,868,915 | 11,107 | 195,865 | 335,236 | 239,389 | 6,087,318 | 875,565 | 5,211,753 | 875,565 | 2,649,035 | 3,524,600 | 0.34 |
| 1003 | 6,944,903 | 11,344 | 199,289 | 341,519 | 244,482 | 6,148,269 | 891,669 | 5,256,600 | 891,669 | 2,672,103 | 3,563,772 | 0.34 |
| 1002 | 6,642,855 | 11,299 | 188,202 | 339,398 | 246,936 | 5,857,020 | 1,044,208 | 4,812,812 | 1,044,208 | 2,447,418 | 3,491,626 | 0.31 |
| 1161001b | 6,594,383 | 10,277 | 183,381 | 319,372 | 228,920 | 5,852,433 | 824,632 | 5,027,801 | 824,632 | 2,556,695 | 3,381,327 | 0.33 |

***, Nuclear genome (780 Mb) used as reference for genetic variation and heterozygosity analysis**

****, Homozygous sites identified possibly due to errors in the reference genome**

**^1^, Filtered at 100% variant frequency**

**^2^, Filtered between 25% and 75% of allele frequency**

**^3^, Percentage of heterozygous SNP positions in a genome of 780 Mb**

Table S7: Polymorphic sites in up to seven of the eight *Macadamia jansenii* accessions

| **Accession ID** | **Heterozygous polymorphic sites (Group A)^1^** | **Heterozygous polymorphic sites (Group B)^2^** | **Total heterozygous polymorphic sites (Group A + B)** | **Homozygous  polymorphic sites (Group C)^3^** | **Total polymorphic sites** | **As % of positions in the genome of 780 Mb** |
| --- | --- | --- | --- | --- | --- | --- |
| 1005* | 1,496,986 | 752,746 | 2,249,732 | 3070** | 2,252,802 | 0.29 |
| 1161004 | 1,082,034 | 820,671 | 1,902,705 | 111,100 | 2,013,805 | 0.26 |
| 1161003 | 1,184,768 | 1,122,003 | 2,306,771 | 162,541 | 2,469,312 | 0.32 |
| 1161005 | 1,160,412 | 1,029,757 | 2,190,169 | 85,258 | 2,275,427 | 0.29 |
| 1161001a | 1,381,312 | 1,102,897 | 2,484,209 | 109,875 | 2,594,084 | 0.33 |
| 1003 | 903,069 | 1,602,657 | 2,505,726 | 113,837 | 2,619,563 | 0.34 |
| 1002 | 1,153,549 | 1,133,126 | 2,286,675 | 165,048 | 2,451,723 | 0.31 |
| 1161001b | 1,347,285 | 1,046,718 | 2,394,003 | 97,027 | 2,491,030 | 0.32 |
| **All accessions^4^** | 5,253,468 | 1,602,657 | 6,856,125 | 350,149 | 7,206,274 | 0.92 |

***, Nuclear genome (780 Mb) used as reference for genetic variation and heterozygosity analysis**

****, Homozygous sites identified possibly due to errors in the reference genome**

**^1^, Group A, at a given polymorphic position some accessions have either a) a heterozygous variant while the rest have the reference allele or b) all accessions have the heterozygous variants**

**^2^, Group B, at a given polymorphic position some accessions have either a) a heterozygous variant while the rest have a homozygous variant or b) a heterozygous variant while the rest have a homozygous variant or the reference allele**

**^3^, Group C, at a given polymorphic position some accessions have either a) Homozygous variant while the rest have the reference allele or b) all have a Homozygous variant.**

**4, Variants common between genotypes are considered as redundant and removed**

Table S8: Genotype-specific unique polymorphic SNP sites

| **Accession ID** | **Genotype-specific unique polymorphic SNP sites** | | |
| --- | --- | --- | --- |
|  | **Heterozygous** | **Homozygous** | **Total** |
| 1005* | 585,053 | 762 | 585,815 |
| 1,161,004 | 187,441 | 8,615 | 196,056 |
| 1,161,003 | 521,184 | 44,014 | 565,198 |
| 1,161,005 | 608,485 | 40,629 | 649,114 |
| 1161001a | 95,089 | 8,155 | 103,244 |
| 1,003 | 99,715 | 9,878 | 109,593 |
| 1,002 | 219,933 | 27,408 | 247,341 |
| 1161001b | 83,662 | 4,843 | 88,505 |
| **All genotypes** | **2,400,562** | **144,304** | **2,544,866** |

***, Nuclear genome (780 Mb) used as reference for genetic variation and heterozygosity analysis**

****, Homozygous sites identified possibly due to errors in the reference genome**

**^1^, Variant sites only found in this individual and not in any of the other 7 genotypes**

Table S9: List of types of duplicate genes in *M. jansenii* genome, using McScanX tool.

| **Type of duplication** | **Number** |
| --- | --- |
| WGD/Segmental | 6303 |
| Tandem | 2966 |
| Proximal | 2573 |
| Dispersed | 15875 |
| Singleton | 3183 |

**Supplementary figures:**


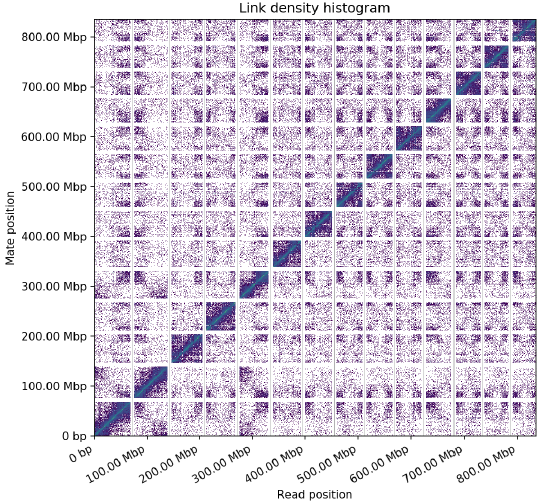


Figure S1: Linkage density histogram of Hi-C assembly of *M. jansenii* genome

The x and y axes give the mapping positions of the first and second read in the read pair respectively, grouped into bins. The colour of each square gives the number of read pairs within that bin. White vertical and black horizontal lines have been added to show the borders between scaffolds. Scaffolds less than 1 Mb are excluded.

**
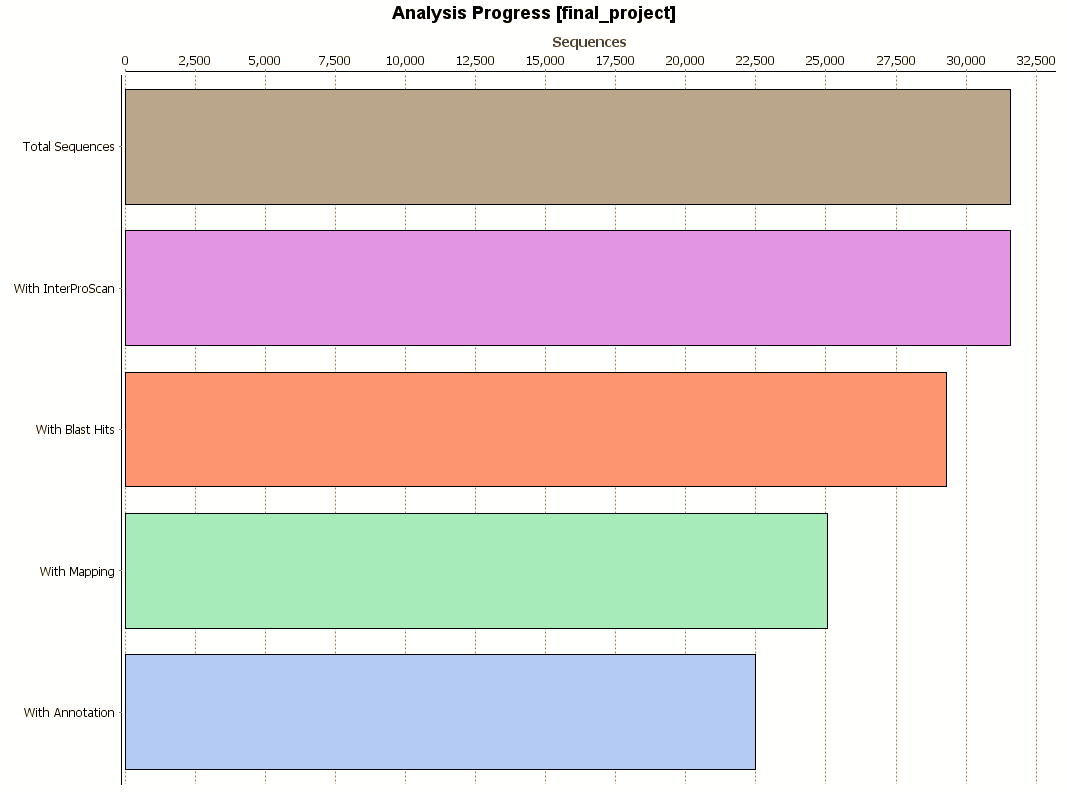
**

Figure S2**:** BLAST2GO sequence similarity search

**
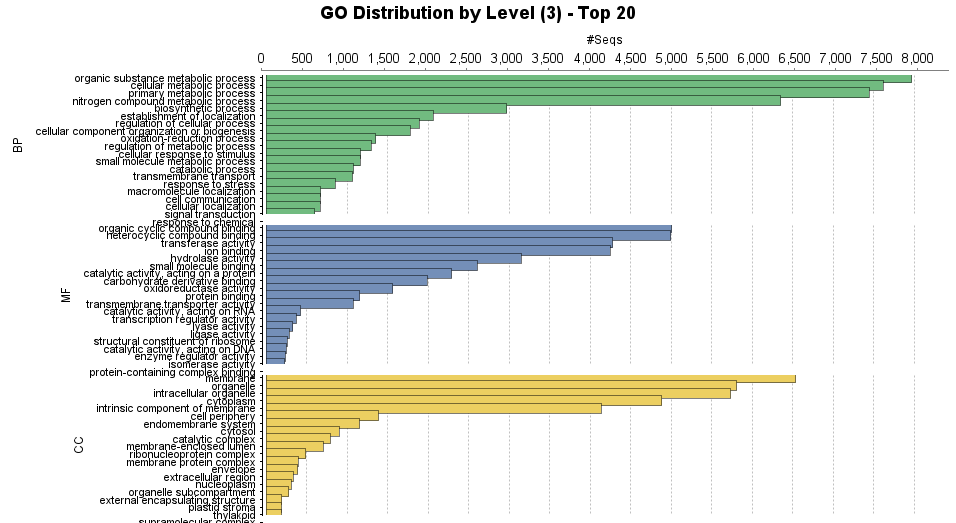
**

Figure S3: Gene ontology (GO) analysis by BLAST2GO


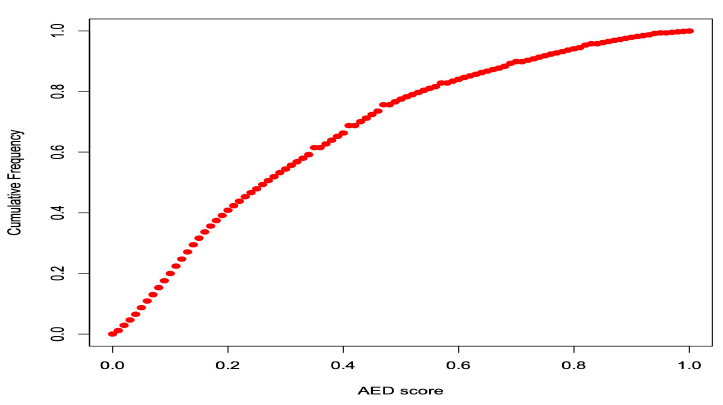


Figure S4: Frequency graph of AED scores.

Annotation edit distance (AED) is a general measure of how well the predicted gene is supported by external evidence (UniProt protein and mRNA sequences). AED score ranges from 0 to 1 and a lower score represents more evidence support for the gene. AED is calculated for every gene. The AED cumulative frequency graph above provides an overview of the quality of the gene annotation.


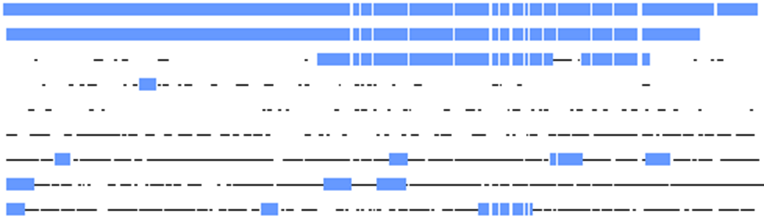


**A**


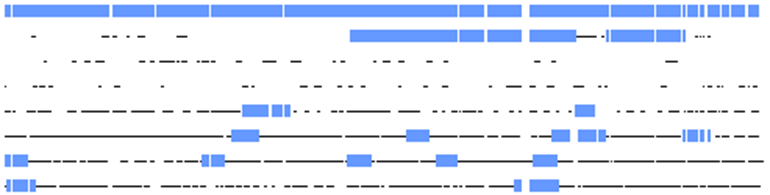


**B**

Figure S5: Alignment of the vicilin-like antimicrobial-peptide transcript from *M*. *integrifolia* and *M. jansenii*

**Figure S5 (A):** MiAMP-2, antimicrobial cDNA sequence from *Macadamia integrifolia*; ANN01396, ANN01395, ANN01396, ANN08351, ANN02865, ANN23835, ANN15809, ANN09965, transcripts identified from the functional annotation of the *Macadamia jansenii* assembly. The MiAMP-2 sequence show high homology to two of the *M. jansenii* transcripts, ANN01396 and ANN01394. **Figure S5 (B):** The *M. jansenii* transcript ANN01396 shown partial homology to the ANN01394 transcript mainly at the second half of the sequence.


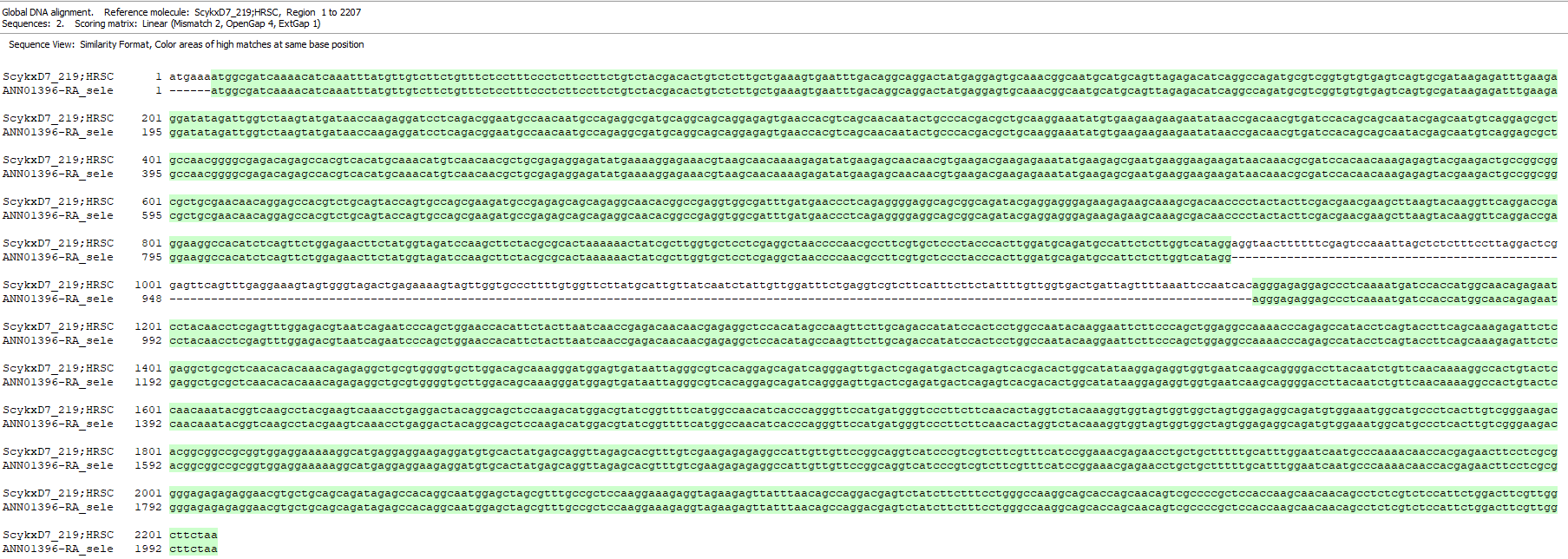


Figure S6: Alignment of anti-microbial CDS sequence of *M. integrifolia* against the *M. jansenii* transcript sequence. The gap in between depicts the intron position in the gene.


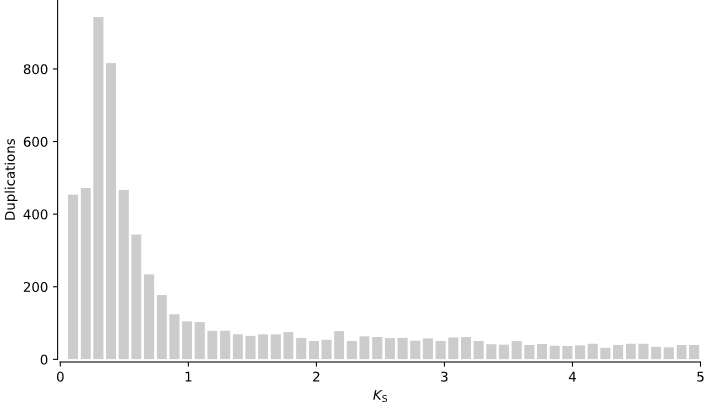


Figure S7: Ks plot of *M. jansenii* was generated by Wgd tool. The plot shows a peak at 0.3, which indicates only one whole genome duplication event in this species.
